# A commercial bacteriophage cocktail failed to decolonize *Zophobas morio* larvae and promoted overgrowth of an OXA-48-producing *Salmonella enterica*

**DOI:** 10.1007/s10096-025-05275-6

**Published:** 2025-10-23

**Authors:** Claudia Aldeia, Edgar I. Campos-Madueno, Andrea Endimiani

**Affiliations:** Institute for Infectious Diseases, University of Bern, Friedbühlstrasse 25, Bern, CH-3001 Switzerland

**Keywords:** *Salmonella*, Gut, Bacteriophages, *Zophobas*, Carbapenemase, Microbiota

## Abstract

**Purpose:**

Effective decolonization strategies for intestinal carriers of carbapenem-resistant Enterobacterales are essential to prevent severe life-threatening infections. In this work, we established gut colonization in *Zophobas morio* larvae (*Zm*L) using an OXA-48-producing *Salmonella enterica* ST198 strain (*Sk*-1) and assessed the commercial *INTESTI* bacteriophage cocktail (*INTESTI*bc) for decolonization.

**Methods:**

*Zm*L were fed with food contaminated with *Sk*-1 (*INTESTI*bc-susceptible) for 3 days and then maintained on a non-contaminated diet until day 14 (T14). At T3, *Zm*L were grouped in untreated, dPBS- or *INTESTI*bc-treated (oral force-feeding on T3 and T5). At specified intervals, *Zm*L were sampled for quantification and characterization of *Sk*-1 (antibiotic/*INTESTI*bc susceptibility and whole-genome sequencing). *Zm*L microbiota was also investigated by 16S rRNA amplicon sequencing.

**Results:**

*Zm*L were rapidly colonized by *Sk*-1 across all groups (T3: 4.3 × 10^6^ CFU/mL). Untreated and dPBS-treated larvae remained consistently colonized (T10: 3.4–9.1 × 10^4^ CFU/mL; T14: 2.9–5.9 × 10^4^ CFU/mL), whereas *INTESTI*bc treatment induced a significant *Sk*-1 regrowth (T10: 4.0 × 10^6^ CFU/mL; *P* < 0.05 vs. controls). *Sk*-1 strains recovered under different conditions between T7 and T14 did not show phenotypic and genotypic changes. Bacteriophages administration resulted in reduced relative abundance of potential bacterial competitors of *Sk*-1 (i.e., *Pseudocitrobacter*).

**Conclusions:**

*Zm*L can be used as a new in vivo model of intestinal colonization with *S. enterica*. However, *INTESTI*bc administration failed to achieve decolonization and instead promoted hazardous overgrowth of the inoculated pathogen. These findings highlight the need for further investigations to clarify the therapeutic potential or possible risks of broad-spectrum bacteriophage cocktails against intestinal infections/colonization caused by hyperepidemic *S. enterica* clones.

**Supplementary Information:**

The online version contains supplementary material available at 10.1007/s10096-025-05275-6.

## Introduction

*Salmonella enterica* isolates are important pathogens primarily responsible for gastroenteritis, but may also cause invasive infections with high mortality rates. In this context, third-generation cephalosporins (3GCs) and fluoroquinolones are usually used for treatment [1–3]. However, the high prevalence of 3GC-resistant *S. enterica* strains that produce plasmid-mediated extended-spectrum β-lactamases (ESBLs) or AmpC represent a serious concern [4–6]. Even more alarming is the recent emergence of global hyperepidemic clones, such as sequence type (ST) 198 serovar Kentucky [7–11], which are capable of producing carbapenemase enzymes (e.g., OXA-48-like, KPC- and NDM-types) [12–15]. Overall, these 3GC-/carbapenem-resistant *S. enterica* strains can lead to difficult-to-treat infections, but they may also contaminate the food chain and colonize the intestinal tract of both humans and animals [9, 16, 17], contributing to their further spread and expansion among different settings [18, 19].

As for other Enterobacterales, finding strategies for decolonizing gut carriers of multidrug-resistant (MDR) *S. enterica* is therefore a public-health priority [20]. In the past, several approaches (e.g., bacteriophages, fecal microbiota transplantation, probiotics) have been explored and envisioned. Nevertheless, such suggestions were based on limited data that were mostly obtained with difficult to implement animal models (e.g., mouse, chicken) [21–24]. For instance, the gold-standard mouse model presents severe ethical (e.g., pain experienced by animals) and logistical limitations (e.g., requirement for skilled personnel, designated facilities), which can all together generate expensive and time-consuming investigations. In contrast, the use of an invertebrate model could provide a cheap, suitable and highly scalable alternative in agreement with the Replacement, Reduction and Refinement (3Rs) framework [25]. Such a model may also be useful for rapid in vivo testing of novel treatment and decolonization strategies, generating detailed preclinical information prior to human clinical trials.

*Zophobas morio* is an insect that belongs to the family of *Tenebrionidae* beetles. Its late-instar larvae (700 ± 50 mg and 45 ± 5 mm in length) possess a hard exoskeleton and may remain in this stage without pupating for > 6 months. Moreover, *Z. morio* larvae (*Zm*L) can be easily reared on a wide range of diets [26]. This last aspect determines the unusual richness and diversity of bacterial species that can populate their gut, resembling that of humans [27, 28]. Overall, due to these characteristics, *Zm*L could provide an advantageous model compared to other invertebrates (e.g., *Galleria mellonella*, *Gm*L) for simulating the gut colonization [29].

Recently, we designed a new intestinal colonization model using *Zm*L [27]. We demonstrated that larvae could be persistently colonized with MDR *Escherichia coli* isolates belonging to hyperepidemic clones (e.g., ST131 producing the CTX-M-15 ESBL, ST410 producing the OXA-181 carbapenemase) after being fed contaminated food. We also showed that the use of the commercial *INTESTI* bacteriophage cocktail (*INTESTI*bc; Eliava BioPreparations) was able to fully decolonize *Zm*L when the MDR *E. coli* was *INTESTI*bc-susceptible in vitro [27].

In this work, *Zm*L were successfully colonized by a hyperepidemic carbapenemase-producing *S. enterica* strain (*Sk*−1) and then treated with the *INTESTI*bc. As a result, though *Sk*−1 was *in vitro INTESTI*bc-susceptible, the in vivo model showed that the bacteriophage treatment was ineffective, leading instead to an unexpected overgrowth of the pathogen. Microbiota analyses also revealed that *INTESTI*bc treatment resulted in a reduction in bacterial richness, including the relative abundance of putative competitors of *Sk*−1.

## Materials and methods

### Features of the colonizing strain (Sk-1)

*Sk*−1 was isolated in 2012 from a perianal screening culture of a patient. It was previously identified as a *S. enterica* subsp. *enterica* serovar Kentucky of ST198 possessing several antimicrobial resistance genes, including the *bla*_OXA−48_ carbapenemase- (IncL plasmid) and the *bla*_VEB−8_ ESBL- (chromosomally located) encoding genes [12]. Antimicrobial susceptibility testing (AST) performed with the Sensititre^™^ MIC microdilution ESB1F and GNX2F panels (Thermo Fisher Scientific) indicated that *Sk*−1 was resistant to all cephalosporins, piperacillin-tazobactam, ciprofloxacin, amikacin, tobramycin and ertapenem (Table 1) [12].

**Table 1:** Phenotypic and molecular characteristics of the original *S. enterica* subsp. *enterica* serovar Kentucky strain *Sk*−1 used for *Zm*L experiments

Antibiotic	MIC, µg/mL (interpretation)^a^
Ampicillin	> 16 (R)
Piperacillin-tazobactam	> 64 (R)
Ticarcillin-clavulanate	> 128 (R)
Cefazolin	> 16 (R)
Cephalothin	> 16 (NA)
Cefpodoxime	> 32 (R)
Cefoxitin	8 (NA)
Ceftriaxone	128 (R)
Cefotaxime	> 64 (R)
Cefotaxime-clavulanate	32 (NA)
Ceftazidime	> 128 (R)
Ceftazidime-clavulanate	> 128 (NA)
Cefepime	> 16 (R)
Aztreonam	> 16 (R)
Imipenem	≤ 0.5 (S)
Meropenem	≤ 1 (S)
Ertapenem	1 (R)
Doripenem	0.25 (S)
Gentamicin	≤ 1 (S)
Tobramycin	> 8 (R)
Amikacin	16 (R)
Ciprofloxacin	> 2 (R)
Levofloxacin	> 8 (R)
Trimethoprim/sulfamethoxazole	≤ 0.5 (S)
Colistin	≤ 0.25 (S)
Polymyxin B	0.5 (NA)
Minocycline	8 (NA)
Doxycycline	8 (NA)
Tigecycline	0.5 (S)
Susceptibility to *INTESTI* bacteriophage cocktail	++++ (S)
	*bla*_OXA−48_ (β-lactams)
	*bla*_VEB−8_ (β-lactams)
	*aac(6)-Ib* (aminoglycosides)
	*tet*(A) (tetracyclines)
	*sul1* (sulfonamides)
Antimicrobial resistance genes (targeted antibiotics)^b^	*mphA* (macrolides)
	*#1: ~63.5-Kb* *- IncL*(*bla*_OXA−48_)
	*#2:* * ~* *5.7-Kb* * - * *Col156*
	*#3:* * ~* *2.1-Kb* * - * *Col(MP18)*
Plasmids	#4: ~2-Kb - ColpVC
Sequence type (ST)	ST198

^a^According to the EUCAST criteria 2025 (v15): R, resistant; S, susceptible; NA, not applicable or not available. MIC values were obtained implementing the Sensititre ESB1F and GNX2F plates (Thermo Fisher Scientific)

^b^*Sk*-1 also had amino acid substitutions in GyrA (Ser83Phe and Asp87Asn) and ParC (Ser80Ile) conferring resistance to fluoroquinolones

In the present study, susceptibility of *Sk*−1 to the *INTESTI*bc was tested using the double-layer agar method (DLA) [30]. *Sk*−1 also underwent whole-genome sequencing (WGS) with both Illumina NovaSeq 6000 (2 × 150 bp) and Nanopore MinION^™^ Mk1B (SQK-RBK004 rapid barcoding kit; FLO-MIN106D R9.4.1 flow cell, 48 h) technologies, as previously described [31, 32]. Briefly, short- and long-read adapter sequences were trimmed using Trimmomatic v0.39 and Porechop v0.2.4, respectively. Hybrid assembly was generated with Unicycler v0.5.0 and polished one round using Polypolish v0.6.0 and PyPolca v0.3.1. The full nucleotide dataset from the Virulence Factor Database (http://www.mgc.ac.cn/VFs/; downloaded on June 17th, 2025) was used to construct a *Salmonella*-specific database. Virulence factor (VF) genes were investigated by querying the hybrid assembly against the custom database *via* ABRicate v1.0.1 (https://github.com/tseemann/abricate).

### *Zm*L colonization and experimental groups

Late-instar *Zm*L (BUGS-International GmbH) originating from two larval stocks (batch 1: August 2023; batch 2: February 2024) purchased from the same Swiss pet store were used for all experiments, as previously described [27]. Briefly, *Zm*L were reared and kept in polypropylene containers and on a daily diet consisting of fresh pear slices and dry cat food mixed in a substrate of oat flakes (Figure S1). Mortality was recorded as previously defined [33].

**Fig. 1:**
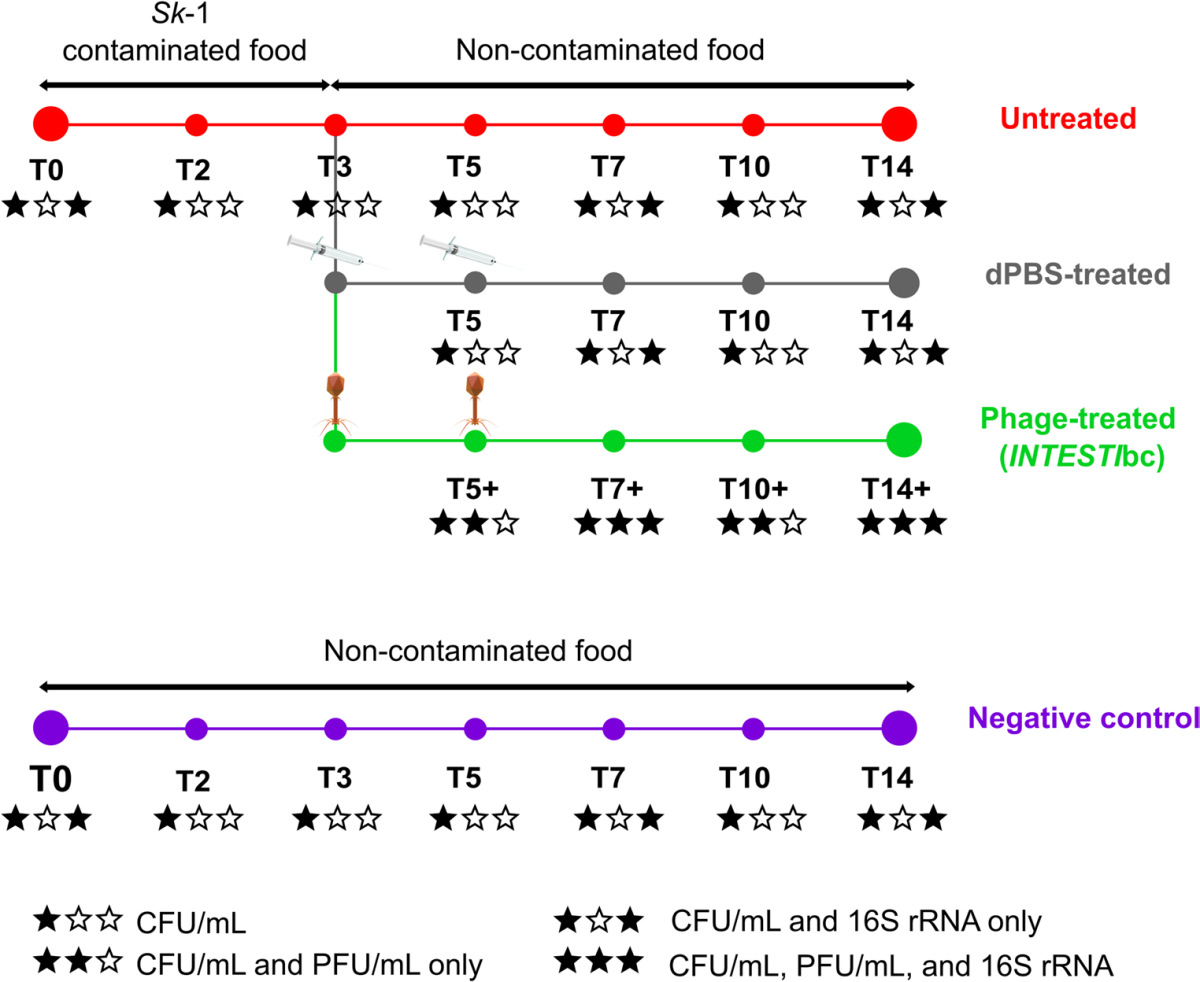
Overall experimental design for the *Zophobas morio* larvae (*Zm*L) assays. T, time point (day). CFU, colony forming unit. PFU, phage forming unit. At T3 and T5, symbols indicate oral administration of either 10 µL dPBS or *INTESTI* bacteriophage cocktail (*INTESTI*bc). Stars below each time point correspond to sampling type (see legend)

To induce gut colonization, *Zm*L were fed for 3 days [from day 0 (T0) to day 3 (T3)] with food contaminated with *S. enterica Sk*−1. To do so, colonies grown on a MacConkey II plate were incubated overnight (36 ± 1 °C) in 10 mL Luria-Bertani (LB) broth. This broth was then poured onto a sterile Petri dish containing the food and incubated (1 h, 36 ± 1 °C) before being given to *Zm*L (Figure S1). After T3, *Zm*L were randomly assigned to 3 experimental groups (i.e., untreated, *INTESTI*bc- and dPBS-treated) and transferred to separate cages, where they received non-contaminated food daily until T14. Three replicates were performed per condition, including three additional experiments for the negative controls that received only non-contaminated food from T0 to T14 (Fig. 1). Notably, experiments with *Zm*L do not require ethical approval (Art. 112 of the Swiss Animal Protection Ordinance; https://www.blv.admin.ch/blv/en/home/tiere/tierschutz.html).

### Treatment with bacteriophages

To attempt decolonizing from *Sk*−1, a randomly selected group of colonized *Zm*L received 10 µL of *INTESTI*bc orally *via* force-feeding on both T3 and T5, administered with a blunt 26s-gauge needle connected to a 250 µL Hamilton 1700 syringe. Another group of colonized *Zm*L was also subjected to force-feeding experiments in which on both T3 and T5 they received 10 µL of sterile 1X Dulbecco phosphate-buffered saline (dPBS) used as control (Fig. 1).

Notably, the *INTESTI*bc [~ 10^5–6^ plaque forming unit (PFU)/mL] targets *E. coli*, *Shigella* spp., *Salmonella* spp. (Paratyphi A, Paratyphi B, Typhimurium, Enteritidis, Cholerasuis, Oranienburg), *Proteus vulgaris/mirabilis*, *Pseudomonas aeruginosa*, *Staphylococcus aureus*, and *Enterococcus* spp [34]. All experiments were conducted using the same *INTESTI*bc lot (M2-1301).

### Analysis of *Zm*L

*Zm*L were processed as previously described [27] (Fig. 1). In brief, for each experiment, 4 *Zm*L were randomly sampled at 7 time points (Tps): T0 (before administering contaminated food), T2, T3 and T5 (both before the corresponding force-feeding with *INTESTI*bc or dPBS), T7, T10, and T14. For simplicity, samples taken during experiments with the use of *INTESTI*bc will be hereafter referred to as T5+, T7+, T10+, and T14+. For each Tp, the 4 *Zm*L were placed inside a 50 mL Falcon tube, euthanized (1 h, −20 °C), and then disinfected (70% ethanol, 3 h, 4 °C). After that, *Zm*L were homogenized using the Precellys^®^ Evolution Touch tissue apparatus (Bertin Technologies) [27].

To detect *Sk*−1, final homogenized samples were properly diluted in dPBS and 100 µL aliquots were plated on selective ChromID^®^ ESBL agar (bioMérieux) that were incubated overnight (36 ± 1 °C). After species identification using the MALDI-TOF MS (Brucker), colony forming unit (CFU)/mL counting for *Sk*−1 was performed for all the Tps.

Bacteriophage titers were evaluated using the DLA method, as previously done [27, 35]. In brief, homogenized samples from *INTESTI*bc*-*treated *Zm*L were filtered using a 0.22 μm pore size PES syringe (Carl Roth Gmbh). Brain Heart Infusion (BHI) agar (1.5%) was distributed as a first layer in a Petri dish. Then, 100 µL of the filtrated tissues was added to a 15 mL tube containing 1 mL of BHI broth. Subsequently, 100 µL of the previously characterized *INTESTI*bc-susceptible *E. coli* strain 56-M3-*Ec* (++++; confluent lysis [36]) or *Sk*−1 were combined with 4 mL of 0.6% agar BHI overlay and poured over the surface of a dried underlay plate. Upon an overnight incubation (36 ± 1 °C), plaques (if any) were counted to determine viral titers (PFU/mL) from samples obtained at T3 (null control), T5+, T7+, T10+, and T14+.

### Characteristics of *Sk*-1 strains recovered over time

Selected *Sk*−1 strains (*n* = 12) recovered during *Zm*L experiments across multiple Tps and experimental conditions underwent AST and DLA assays. Results were compared to those obtained from the original *Sk*−1 strain.

The 12 strains also underwent hybrid WGS. gDNA was extracted using the PureLink™ Microbiome DNA Purification kit (Thermo Fisher Scientific). WGS was performed on a NovaSeq 6000 (Illumina; 2 × 150 bp paired-end protocol), and MinION™ (Nanopore; SQK-RBK114.24 rapid barcoding kit 24 V14, FLO-MIN114 R.10.4.1 flow cell, 72 h) sequencing platforms. Read pre-processing and the pipeline used to generate the hybrid assemblies were performed as described above. Hybrid genome assemblies were used to generate whole-genome single nucleotide variants (SNVs) alignments (chromosome and plasmids) with snippy v4.6.0, using the original *Sk*−1 genome as a reference, as previously described [31, 32, 37]. The input assemblies were further analyzed for structural variations implementing SyRI v1.7.1 using paired (vs. reference) genome filtered alignments (delta-filter: ‘- 90 -l 100’) generated with nucmer v3.1 and the ‘--maxmatch’ argument.

### Microbiota characterization

Microbiota characterization for *Zm*L was achieved through 16S rRNA amplicon sequencing analysis as previously done [27]. Samples obtained at T0, T7/T7+, and T14/T14 + for *Zm*L were analyzed (Fig. 1).

Briefly, gDNA was isolated from the homogenized tissues of *Zm*L using the QIAamp PowerFecal Pro DNA Kit (Qiagen) and purified with CleanNGS magnetic beads (CleanNA). DNA concentration and purity were assessed with Qubit™ 3.0 and NanoDrop™ One^C^ (Thermo Fisher Scientific), respectively. gDNA preparations were sent to Eurofins Genomics GmbH (Germany) for amplification and sequencing of the bacterial 16S rRNA gene (V4 region). Genomic libraries were prepared using a PCR amplification with the primers 515 F Parada and 806R Apprill [38]. All samples were sequenced on an Illumina MiSeq platform (2 × 300 bp read lengths) and the resulting read quality was assessed with FastQC v0.12.1.

Raw sequence data in FASTQ format were processed with the DADA2 pipeline v1.22.0 in R programming language v4.4.2 [parameters: truncLen f/r 235/190, maxN = 0, maxEE = c(2,2), truncQ = 2, rm.phix = TRUE, compress = TRUE, and multithread = TRUE] using the SILVA reference database v138.1 Nr99 as previously described [27, 39]. Agglomerated amplicon sequence variants (ASVs) at the genus level and their relative abundances were determined using the phyloseq v1.50.0 R package.

Alpha diversity metrics [Shannon diversity index (SDI) and Observed richness (S_obs_)] were calculated on non-normalized ASV counts with phyloseq. Additionally, differentially abundant genera were assessed using the DESeq2 v1.46.0 R package, adjusted for batch variation, and applied on non-normalized ASV counts, specifying comparisons within-groups across time points (*P*-adj < 0.05) *via* the contrast argument. Microbial compositional variation was determined after cumulative-sum scaling (CSS) normalization from metagenomeSeq v1.48.1 to consider differences in sequencing depth, while further batch-to-batch correction was performed with ConQuR v2.0 with a penalized fitting strategy. Beta diversity was assessed using the Bray-Curtis dissimilarity index (BCDI) and principal coordinate analysis (PCoA) of the Bray-Curtis distance matrix with phyloseq.

### Statistical analyses

Statistical analyses were performed using R v4.4.2 as previously done [27, 33]. Data normality and homogeneity of variances was assessed using the Shapiro-Wilk and Levene’s tests. Growth curves (CFU/mL) were statistically compared using a two-way ANOVA on log-transformed data (log_10_) to assess the interaction between experimental conditions and time points (stats package v4.4.2). *Post hoc* comparisons were carried out with estimated marginal means, while *P* values were adjusted with the Tukey method (emmeans v1.11.1).

For microbiota, alpha diversity was analyzed over time within each experimental condition with the non-parametric Kruskal-Wallis test (stats package v4.4.2). The effect of batch on the community composition at T0 was assessed by permutational multivariate analysis of variance using distance matrices (PERMANOVA, adonis2 function; with 9999 permutations) from the vegan v2.6.10 R package using the Bray-Curtis distance matrix (CSS transformed and batch-corrected). Results were considered statistically significant when *P* value < 0.05. All packages were set to default parameters, unless otherwise indicated. Graphical analyses were visualized with the R package ggplot2 v3.5.2 and modified in Inkscape v1.4 for style.

## Results

### Further data about *Sk*-1

The tested *S. enterica* strain was phenotypically susceptible (“++++”: complete clearing) to the *INTESTI*bc (Table 1). The resulting genome consisted of a circular chromosome (4,841,872-bp) and 4 plasmids (including a ~ 63,5-Kb IncL-*bla*_OXA−48_).

A total of 265 putative VF genes were detected in *Sk*−1 (File S1), including *sseK2* that is linked to biofilm formation and survival under difficult conditions [40]. Concerning the VF genes usually associated with *S. enterica* human infection, only *pipB2* and *sifA* were detected, whereas *Sk*−1 lacked of *grvA*, *sseI*, *sopE*, *sodCI*, *sopD2*, *sspH2*, *srfH*, and *shdA* [17, 41–43].

### *Zm*L colonization and treatment with bacteriophage

**Fig. 2:**
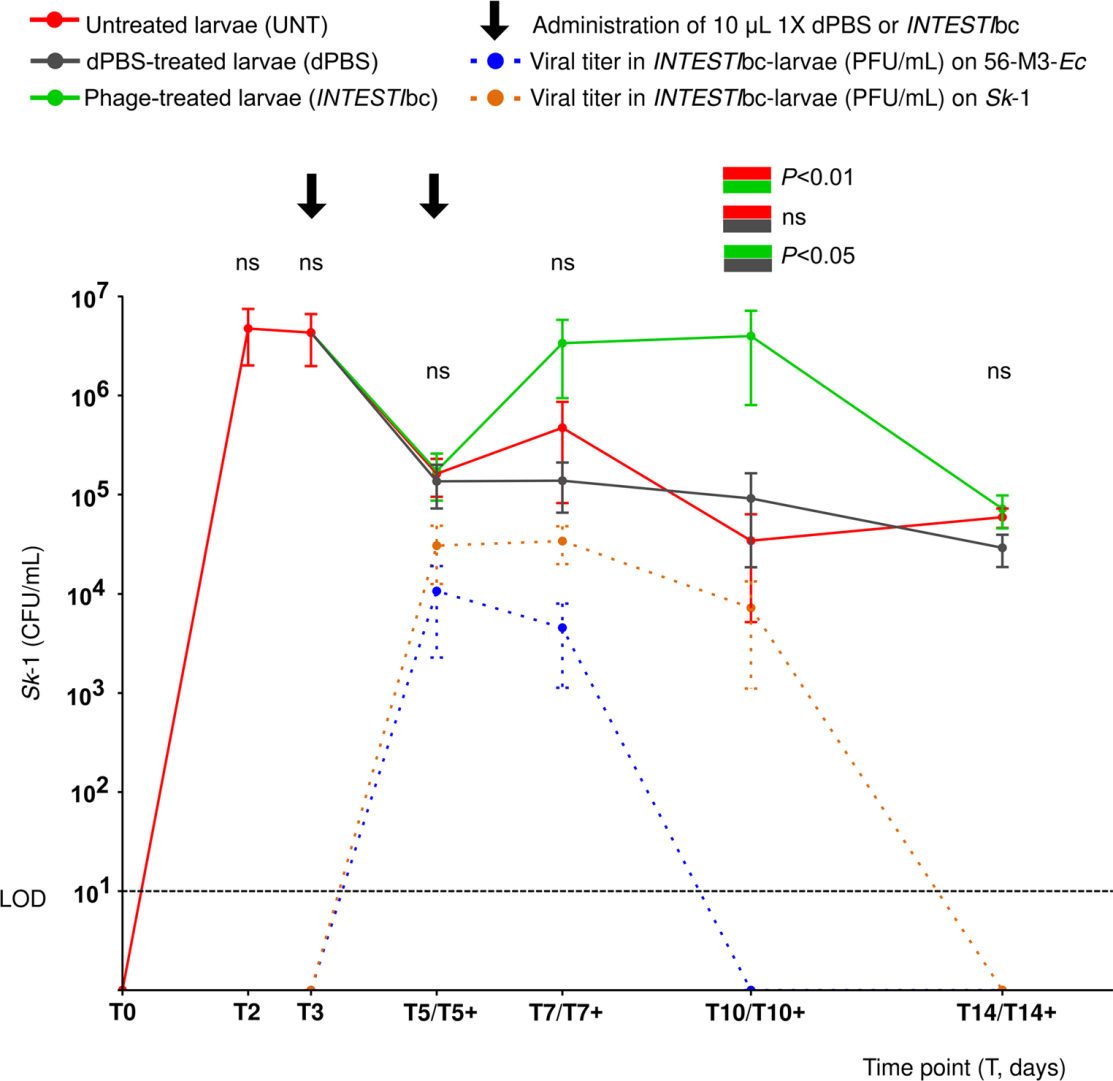
Gut colonization of *Z. morio* larvae (*Zm*L) by *S. enterica* strain *Sk*−1. Y axis: colony forming unit (CFU)/mL or phage forming unit (PFU)/mL [mean ± standard error (SEM; *n* = 3)]. X axis: time points (T, *Zm*L not receiving *INTESTI*bc; T+, *Zm*L receiving *INTESTI*bc). At T3, *Zm*L were randomly assigned to 3 groups: untreated, dPBS-treated and *INTESTI*bc-treated. dPBS and *INTESTI*bc groups received two oral force-feedings (10 µL) at T3 and T5. LOD, limit of detection. ns, not significant

As depicted in Fig. 2 (crude data in Table S1), after providing 3 days of contaminated food, untreated *Zm*L remained persistently colonized with *S. enterica Sk*−1 strain (e.g., mean of 4.3 × 10^6^ and 5.9 × 10^4^ CFU/mL for T3 and T14, respectively). Moreover, administration of dPBS on T3 and T5 did not significantly influence *Sk*−1 load. On the other hand, treatment with two doses of *INTESTI*bc induced a statistically significant overgrowth of *Sk*−1 that reached 4.0 × 10^6^ CFU/mL on T10+ (*P* < 0.01 for *Zm*L treated vs. untreated and *P* < 0.05 vs. the control with dPBS). Finally, bacteriophages targeting *E. coli* 56-M3-*Ec* were recovered on T5 + and T7+ (titers 1.1 × 10^4^ and 4.6 × 10^3^ PFU/mL, respectively), while those against *Salmonella Sk*−1 were detected on T5+, T7+, and T10+ (3.1 × 10^4^, 3.4 × 10^4^, and 7.3 × 10^3^ PFU/mL, respectively).

Notably, *Zm*L mortality after administering food contaminated with *Sk*−1 was 0% at both T3 and T7. Moreover, mortality after force-feeding with *INTESTI*bc or dPBS was 12% and 7% at T7+/T7, respectively (Table S2).

### Dynamics of *Zm*L microbiota

**Fig. 3:**
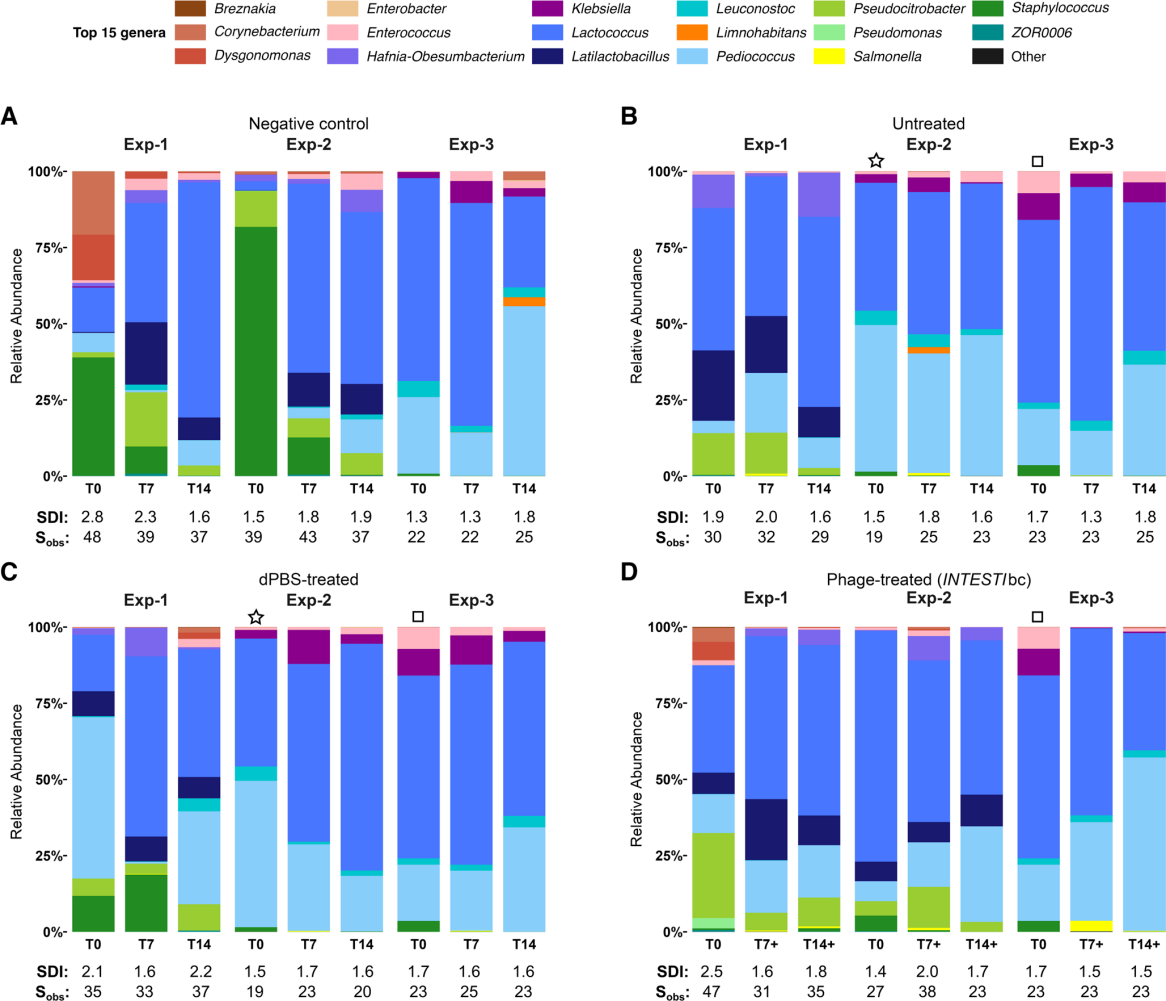
Comparison of the top 15 most abundant genera per sample across experimental groups. Relative community composition of *Zm*L gut microbiota was determined by 16S rRNA gene amplicon sequencing of the V4 region at the genus-level, based on three experiments across time points (T0, T7/T7+, and T14/T14+). Symbols star and square indicate experiments that share the same *Zm*L (T0). Panels show the top 15 genera identified from a total of 26 genera obtained from 292 amplicon sequencing variants (ASVs). All remaining genera are grouped under “Other”. SDI, Shannon diversity index. S_obs_, observed richness

As shown in Fig. 3, the baseline microbiota (before administration of *Sk*−1 at T0) was commonly rich in *Lactococcus*, *Pediococcus*,* Staphylococcus*, and *Latilactobacillus* genera (relative abundance, range: 28–57%, 11–40%, 2–40% and 0.1-8%, respectively). Genera belonging to the *Enterobacteriaceae* family were also present, predominantly represented by *Pseudocitrobacter* (relative abundance, range: 2–11%), while *Escherichia*/*Shigella* were notably absent (File S2). These relative abundance patterns across groups were also supported by differential abundance analysis. In particular, *Lactococcus*,* Pediococcus*,* Staphylococcus*,* Latilactobacillus* and *Pseudocitrobacter* were shown to be significantly decreased at T7 compared to T0 (File S4).

**Fig. 4:**
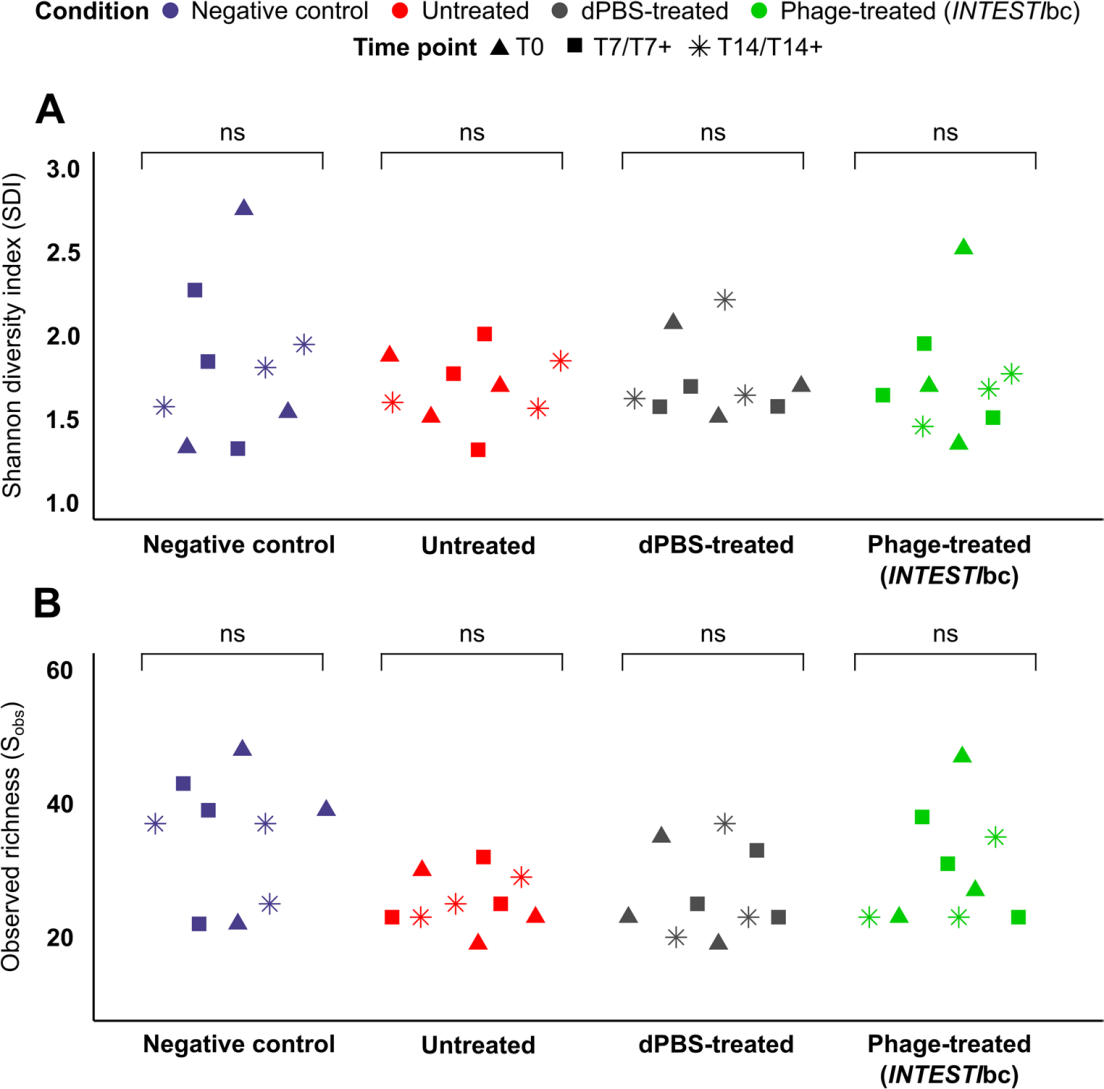
Alpha diversity analysis using the SDI (**A**) and S_obs_ (**B**). Each condition included three experimental replicates (Exp-1, Exp-2, Exp-3), with samples collected at T0, T7/T7 + and T14/T14+. Condition is color-coded and time points are indicated by symbol shape. Above each condition (inverted bracket) ‘ns’ represents no statistically significant differences over time within that group

Alpha diversity analysis indicated that the initial microbiota at T0 was heterogeneous (SDIs and S_obs_ of 1.3–2.8 and 19–48, respectively). Nevertheless, both parameters were not statistically significantly different between the *Zm*L belonging to the same experimental condition and over time (i.e., on T0, T7/T7 + or T14/T14+) (Fig. 4). Remarkably, beta diversity analysis indicated that the microbiota heterogeneity observed at T0 was mainly due to the compositional differences of the two distinct *Zm*L batches utilized for the experiments, as shown by the clear clustering separation by batch in the PCoA ordination (*P* = 0.0128; Figure S2).

After administering the two *INTESTI*bc doses, microbiota patterns showed a decrease in the relative abundance and number of genera (e.g., *Enterococcus*,* Staphylococcus* and *Pseudocitrobacter*), with SDIs and S_obs_ of 1.5–1.8 and 23–35, respectively at T14+. In contrast, it was evident the emergence of the *Salmonella* genus (relative abundance, range: 0.4-3% on T7+ and 0-0.6% on T14+) (Fig. 3D; File S2-F). Differential abundance testing further corroborated these genera-level differences, which identified a strong reduction in abundance of *Enterococcus*, *Staphylococcus* and *Pseudocitrobacter* at T7. On the other hand, a significant increase of *Salmonella* at T7+ and T14+ was recorded (File S4-D). However, alpha diversity analysis showed that these over time microbiota changes were not statistically significant (Fig. 4).

### Features of *Sk*-1 recovered during experiments

Twelve representative *S. enterica Sk*−1 strains were recovered during different *Zm*L experiments at T7/T7+, T10/T10+, and T14/T14+. As summarized in Table S3, all 12 strains demonstrated consistent antibiotic and *INTESTI*bc susceptibility profiles as for the original *Sk*−1.

Genome alignment (98.5%) analysis indicated that each of the 12 strains possessed ≤ 3 SNVs when compared to the initial reference *Sk*−1 genome (Table S4). We also note that the analysis demonstrated small structural changes when compared to the reference. For instance, ≤ 22 and ≤ 21 SNVs and ≤ 3 and ≤ 2 insertions for *Sk*−1 strains recovered during experiments without and with *INTESTI*bc treatment, respectively (File S3).

## Discussion

In agreement with the 3Rs guidelines, insect larvae may represent an alternative to complex animal models for studying the effects of gut bacterial pathogens [25]. In particular, *Gm*L have been used in the past as an infection model to study the virulence of *Salmonella enterica* and potential novel treatment approaches (e.g [44–46]). Nevertheless, the use of *Gm*L for studies focusing on intestinal colonization has limitations. First, *Gm*L possess a very limited microbiota, dominated by *Enterococcus* spp. [47], which does not mimic the richness observed in the human gut. Second, microbiota manipulation *via* contaminated food administration is impracticable, as *Gm*L do not feed voluntarily [29]. Third, *Gm*L may undergo high mortality rates during experiments using *Enterobacteriaceae*, especially if the force-feeding approach is implemented [27, 48]. For instance, Nale JY *et al., *colonized *Gm*L with *S. enterica* strains to demonstrate the therapeutic potential of a three-phage cocktail. However, many optimization experiments were necessary to determine the right oral gavage inoculum for each specific strain to avoid high mortality rates [49].

### *Zm*L were stably colonized by *Sk*-1

In this study, we exploited the unique biological features of *Zm*L to study their susceptibility to colonization by a hyperepidemic ST198 *S. enterica* serovar Kentucky isolate (*Sk*−1) which produces the pandemic OXA-48 carbapenemase [7–15, 26]. As a result, untreated *Zm*L remained stably colonized for 14 days with *Sk*−1 (T14: ~10^4^ CFU/mL) after 3 days of feeding with contaminated food (Fig. 2). We emphasize that this approach resembles natural *Salmonella* infections, which are typically acquired *via* the oral route [1–3, 9]. On the other hand, we did not use the force-feeding administration - as we have previously done with MDR *E. coli* [33] - because it does not mimic the natural acquisition of *S. enterica*.

### *Zm*L underwent low mortality

In the study of Nale YT *et al.*, the mortality rates of untreated *Gm*L that underwent force-feeding with 3 different *S. enterica* isolates (all serovar Typhimurium) were > 80% at 72 h [49]. In contrast, the mortality of untreated *Zm*L colonized with *Sk*−1 was zero at both T5 and T7 (Table S2). This positive effect may be due to *i*) the harmless oral route of administration *via* the diet, *ii*) the lower quantity of pathogen intake compared to the oral gavage, and/or *iii*) the superior physiologic features of *Zm*L compared to those of *Gm*L [26]. Nevertheless, considering the results of the VFs analysis (File S1), it should also be hypothesized that *Sk*−1 is less virulent than the 3 Typhimurium strains tested by Nale YT *et al.* [49]. In particular, the pattern of VF genes detected in *Sk*−1 suggests that the strain is more prone to induce *Zm*L colonization rather than clinical illness [17, 40–43].

The mortality rates of colonized *Zm*L that received two force-feeding administrations (T3 and T5/T5+) with *INTESTI*bc or dPBS were also low (≤ 12% at T7+/T7; Table S2), as we have observed in a previous study [33]. Overall, these findings indicate that *Zm*L are particularly resilient to receive oral injections, supporting the suitability of this model for future experiments of similar design.

### *INTESTI*bc treatment was ineffective

The *INTESTI*bc is a commercially available product used for the treatment or prophylaxis of enteric bacterial infections (*per os*: 10 mL 4 times per day for 5–6 days or 10–20 mL daily every 5 days over the course of 1 month, respectively), including those due to *S. enterica* (https://phage.ge/en/products/phago-intesti) [34]. However, to our knowledge, the activity of this specific bacteriophage cocktail against *S. enterica* colonization/infection and its impact on the microbiota have not yet been evaluated in vivo. Therefore, we used this product in our study to assess its capacity in decolonizing *Zm*L from *S. enterica Sk*−1. Notably, due to potential harm from repeated force-feeding, *Zm*L received only two doses of *INTESTI*bc, as in our previous studies [27, 33]. Nevertheless, we believe that this approach still provides valuable proof-of-concept data that may be translatable to the human clinical context.

In *Zm*L receiving *INTESTI*bc, bacteriophages were detected only between T5+ and T10+ (Fig. 2). We speculate that their absence on T14 + may reflect an inability to locate suitable bacterial hosts for replication and/or because the *Zm*L gut does not provide an optimal environment (e.g., pH, temperature) for viral stability [50]. In this context, the overgrowth of *Sk*−1 between T5+ and T10+ may partly account for the longer persistence of its specific active bacteriophages compared with those targeting *E. coli* 56-M3-*Ec*.

After the first dose of *INTESTI*bc, *Zm*L showed an initial decrease in *Sk*−1 concentration (T5: ~2 × 10^5^ CFU/mL), but an unanticipated regrowth occurred following the second administration and continued through T10+ (~ 4 × 10^6^ CFU/mL), showing a statistically significant difference compared with the control curves (Fig. 2). To explain this phenomenon, we initially hypothesized that *Sk*−1 underwent mutations conferring resistance to bacteriophages, as previously noted by others using mono-phage therapies in *Gm*L colonized with *S. enterica* [24, 44, 49]. However, both phenotypic and genotypic analyses did not support this hypothesis, instead indicating the stability of *Sk*−1 during the 14 days of experiments (Table S3, Table S4 and File S3).

We therefore speculate that the overgrowth of *S. enterica Sk*−1 under *INTESTI*bc treatment was linked to the effect of the overall multiple lytic phages (cocktail) on the complex bacterial population residing within the *Zm*L gut. In particular, we hypothesize that there were specific bacterial competitors of *Sk-1* in the intestinal tract of *Zm*L that could partially protect against its colonization (a phenomenon known as “colonization resistance”) [51]. Since the *INTESTI*bc may inhibit multiple species [34] - including the putative competitors - *Sk-1* could overgrowth in *Zm*L following the peak of bacteriophages observed between T5+ and T7+ (Fig. 2). In this context, microbiota analyses have provided several clues about the possible *Sk*−1 competitors (see below).

### Microbiota underwent small but critical changes

*Zm*L microbiota was characterized over time to assess the effect of *INTESTI*bc on the microbial community in larvae colonized with *S. enterica Sk*−1. Notably, our previous analysis of *Zm*L challenged with MDR *E. coli* strains (with or without administering *INTESTI*bc) revealed a non-significant reduction of the bacterial diversity during experiments [27].

In the present study, the starting *Zm*L microbiota patterns at T0 were heterogeneous, particularly given that two distinct batches of larvae were used in the experiments. Nevertheless, this variation reflects the natural diversity of their microbiota, with many bacterial genera also encountered in the human microbiota (Fig. 3 and Figure S2). Similarly, the human microbiota can vary substantially at the individual level due to various factors (e.g., diverse diet, age, ethnicity, travels) [52–54]. Therefore, these observations suggest that the dynamics of the *Zm*L microbiota recorded under the *INTESTI*bc challenge may mimic those that could occur in the human gut.

As anticipated, administration of *INTESTI*bc to *Zm*L colonized by *Sk*−1 induced significant overgrowth of the pathogen as confirmed by the 16S rRNA microbiota analysis of T7+ and T14+ samples (Fig. 3D, File S4-D). Simultaneously, *INTESTI*bc targets several genera that tend to decrease in abundance - or even disappear entirely - at T7+ and/or T14+. In particular, a specific negative effect against *Pseudocitrobacter* was noted [relative abundance range: T0: 4.69–27.89% vs. T14+: 0.49–9.46% (File S2-F); mean log_2_ fold change range: T7+: −23.6 vs. T14+: −23.7 compared to T0 (File S4-D)]. This genus - belonging to the *Enterobacteriaceae* - has been rarely found in cockroaches and in culture-based fecal samples of humans [well summarized in [55]. Nevertheless, the scarcity of data regarding *Pseudocitrobacter* - including those present in gut microbiome datasets - may just reflect its recent classification. It should also be noted that the routinely used MALDI-TOF MS or VITEK 2 systems may misidentify *Pseudocitrobacter* spp. as *Pantoea* spp. [55].

It should be emphasized that *Enterobacteriaceae* represent < 1% of the total bacteria detected in the gut microbiota of healthy individuals [56]. However, mouse model experiments have demonstrated that these bacteria - especially *E. coli* - play a major role in the colonization resistance against *Salmonella* infections [57, 58]. Therefore, similar to the effects of broad-spectrum antibiotics [20], *INTESTI*bc might negatively affect the competitive activity of some endogenous *Enterobacteriaceae* against *S. enterica* infection. This effect may occur in *Zm*L, animals, or humans, although the specific protective *Enterobacteriaceae* species affected by *INTESTI*bc are likely to differ between hosts (e.g., *Pseudocitrobacter* spp. in *Zm*L vs. *E. coli* in humans).

## Conclusions

In this work, we demonstrated that *Zm*L can be easily and stably colonized with *S. enterica* without affecting larval survival. Therefore, this new in vivo model may represent an inexpensive, simple, fast, and reliable alternative to the gold-standard mouse model for studying several key aspects of this important enteric human pathogen (e.g., development of novel treatment or decolonization strategies) [9, 16, 17, 25].

As a proof-of-concept, we used the *INTESTI*bc to decolonize the *Zm*L carrying an hyperepidemic OXA-48-producing ST198 *S. enterica* [7–15]. Accordingly, the commercial bacteriophages cocktail was ineffective, likely because its disruptive impact on the *Zm*L microbiota led to pathogen overgrowth. This phenomenon may be associated to the inhibition of the *Pseudocitrobacter* genus [55], a member of the *Enterobacteriaceae* which appears not significantly present in the human microbiota, but may confer a protective effect against *S. enterica* colonization in *Zm*L.

Notably, the overgrowth of *Sk*−1 observed with our in vivo model was transient (i.e., the *Sk*−1 load returned to baseline at T14+) following the two consecutive *INTESTI*bc administrations. However, it is plausible that during prolonged treatment in humans or animals (e.g., 5–6 days as suggested) [34], repeated *INTESTI*bc doses may continue to promote the surplus of the pathogen at the intestinal level.

Regardless of host-specific differences in *Enterobacteriaceae* species that mediate colonization resistance, further in vivo studies are needed to assess the suitability of using bacteriophages to treat human/animal intestinal infections or colonization due to hyperepidemic MDR clones of *S. enterica*. In fact, while the mono-phage treatment is at risk of selecting a resistant phenotype [24, 44, 49], our results suggest that the use of broad-spectrum cocktails could promote a dangerous overgrowth of the targeted enteric pathogen. Therefore, the development and use of commercial formulations containing several lytic bacteriophages targeting only the specific enteric pathogen is envisioned for future in vivo studies followed by clinical trials [24, 44, 49].

## Supplementary Information

Below is the link to the electronic supplementary material.

Supplementary Material 1

## Data Availability

The complete genome sequence of *S.* *enterica* *Sk*-1 (reference) has been deposited under BioProject accession number PRJNA1267977. The complete genome assemblies of the 12 *Sk*-1 strains recovered during experiments have been deposited under BioProject number PRJNA1267977. The raw 16 S rRNA sequencing data has been deposited under BioProject accession number PRJNA1268327.
