# Supplementary material for: A commercial bacteriophage cocktail failed to decolonize Zophobas morio larvae and promoted overgrowth of an OXA-48-producing Salmonella enterica
Source: Eur J Clin Microbiol Infect Dis. 2025 Oct 23;45(2):363–74. doi: 10.1007/s10096-025-05275-6 (PMC12987905; doi:10.1007/s10096-025-05275-6)
Supplement: Supplementary file 1 — Supplementary Material 1 [file 10096_2025_5275_MOESM1_ESM.pdf]

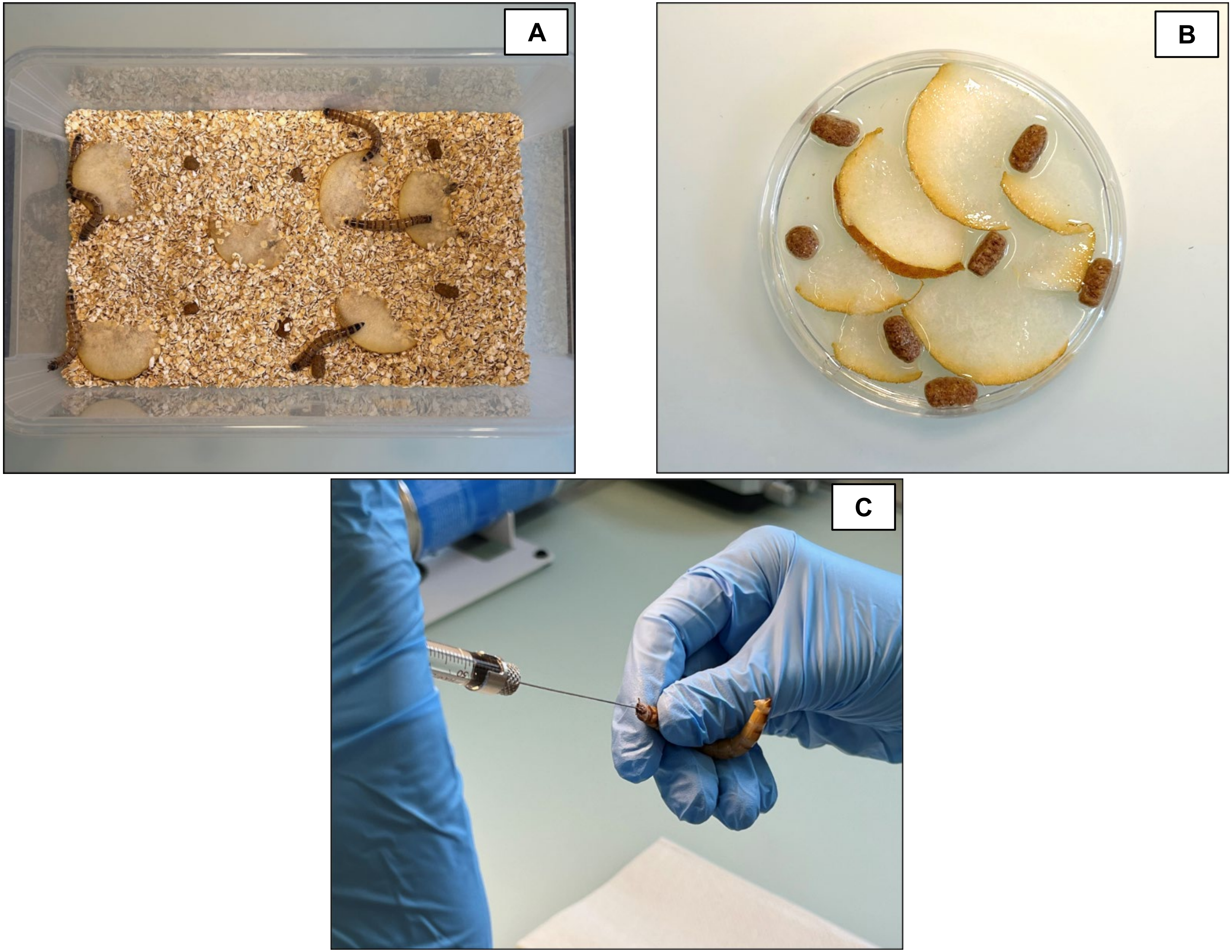

Figure S1. *Zophobas morio* larvae (*Zm*L) rearing and colonization with *S. enterica Sk*-1. **A)** Plastic container, including food source and *Zm*L. **B)** Petri dish containing contaminated food overnight in LB broth; dishes are incubated for 1h at 36±1 °C and subsequently distributed over the oat substrate. **C)** Force-feeding: larvae are immobilized in a fixed position and a 26s-gauge needle is directly inserted into the mouth for oral injection with either 10 µL of 1X dPBS or *INTESTI* bacteriophage cocktail.

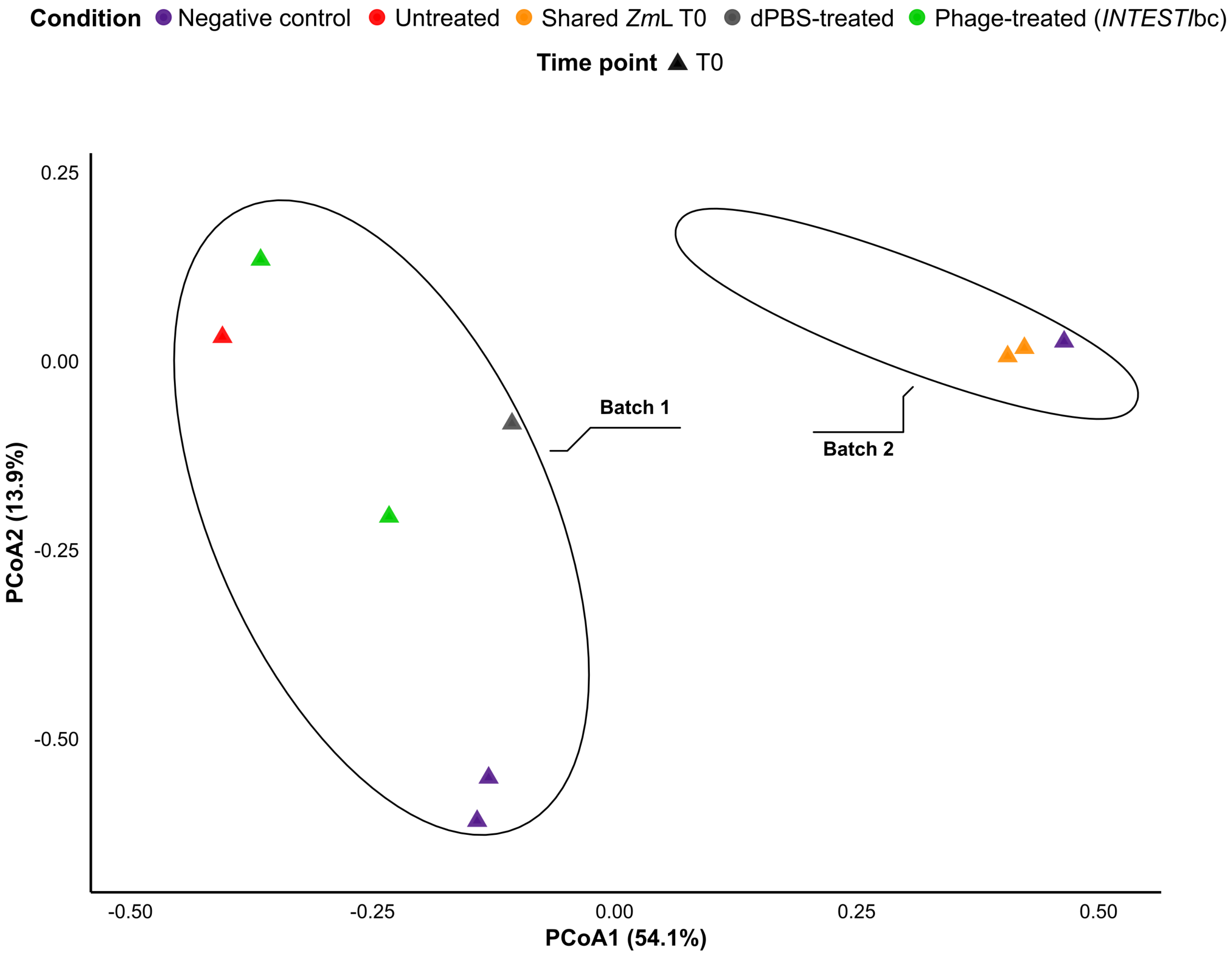

Figure S2. Beta diversity using principal coordinate analysis (PCoA) based on the Bray-Curtis distance matrix, illustrating the composition of bacterial communities at the start time point (T0; n=9 samples). Adult *Zm*L from batch 1 were experimentally available in August 2023, whereas those from batch 2 in February 2024. Each sample is colored by condition and ellipses are drawn around each batch group. The taxonomic composition of the *Zm*L microbiota at T0 differs significantly between batches, as indicated by permutational multivariate analysis of variance ($\text{PERMANOVA}_{9999\ \text{permutations}}$, F= 4.80, R^2= 0.41, P=0.0128). PCoA1 and PCoA2 explained 54.1% and 13.9% of the variance, respectively.

Table S1. Data from all *Z. morio* larvae *in vivo* experiments expressed in colony-forming units (CFU)/mL or plaques-forming units (PFU)/mL

***Zm*L Group**	**Experiment #**	**CFU/mL or PFU/mL**						
		**T0**	**T2**	**T3**	**T5**	**T7**	**T10**	**T14**
**Untreated**	Exp1	0	$6.27x10^{4}$	$1.46x10^{7}$	$3.08x10^{4}$	$1.25x10^{6}$	$7.35x10^{3}$	$3.29x10^{4}$
	Exp2	0	$1.68x10^{7}$	$1.38x10^{6}$	$2.53x10^{5}$	$1.00x10^{5}$	$9.21x10^{4}$	$7.30x10^{4}$
	Exp3	0	$8.63x10^{6}$	$3.59x10^{4}$	$2.02x10^{5}$	$6.41x10^{4}$	$3.18x10^{3}$	$7.15x10^{4}$
	**Mean**	**0** [a]	**$4.74x10^{6}$** [a]	**$4.31x10^{6}$** [a]	**$1.62x10^{5}$**	**$4.72x10^{5}$**	**$3.42x10^{4}$**	**$5.91x10^{4}$**
**Phage-treated (*INTESTI*bc)**	Exp1	0	$1.20x10^{6}$	$5.83x10^{5}$	$8.48x10^{4}$	$4.64x10^{5}$	$6.16x10^{5}$	$9.47x10^{4}$
	Exp2	0	$9.77x10^{5}$	$1.77x10^{6}$	$8.86x10^{4}$	$1.45x10^{6}$	$9.82x10^{5}$	$1.94x10^{4}$
	Exp3	- [b]	- [b]	- [b]	$3.45x10^{5}$	$8.19x10^{6}$	$1.03x10^{7}$	$1.01x10^{5}$
	**Mean**	- [b]	- [b]	- [b]	**$1.73x10^{5}$**	**$3.37x10^{6}$**	**$3.98x10^{6}$**	**$7.17x10^{4}$**
**dPBS-treated**	Exp1	0	$8.27x10^{5}$	$7.51x10^{6}$	$2.84x10^{4}$	$3.14x10^{4}$	$4.10x10^{3}$	$8.78x10^{3}$
	Exp2	- [b]	- [b]	- [b]	$2.49x10^{5}$	$1.07x10^{5}$	$2.36x10^{5}$	$4.27x10^{4}$
	Exp3	- [b]	- [b]	- [b]	$1.31x10^{5}$	$2.76x10^{5}$	$3.42x10^{4}$	$3.57x10^{4}$
	**Mean**	- [b]	- [b]	- [b]	**$1.36x10^{5}$**	**$1.38x10^{5}$**	**$9.13x10^{4}$**	**$2.90x10^{4}$**
**Viral titer on 56-M3-*Ec***	Exp1	NP	NP	0	$2.75x10^{4}$	$1.14x10^{3}$	0	0
	Exp2	NP	NP	0	$1.14x10^{3}$	$1.14x10^{4}$	0	0
	Exp3	NP	NP	0	$3.43x10^{3}$	$1.14x10^{3}$	0	0
	**Mean**	**NP**	**NP**	**0**	**$1.07x10^{4}$**	**$4.58x10^{3}$**	**0**	**0**
**Viral titer on *Sk*-1**	Exp1	NP	NP	0	$6.29x10^{4}$	$6.86x10^{3}$	0	0
	Exp2	NP	NP	0	$1.14x10^{3}$	$4.23x10^{4}$	$1.94x10^{4}$	0
	Exp3	NP	NP	0	$2.75x10^{4}$	$5.26x10^{4}$	$2.29x10^{3}$	0
	**Mean**	**NP**	**NP**	**0**	**$3.05x10^{4}$**	**$3.39x10^{4}$**	**$7.25x10^{3}$**	**0**

Note. *INTESTI*bc, *INTESTI* bacteriophages cocktail; NP, not performed.

[a] Mean CFU/mL represent six biological replicates across the three treatment groups.

[b] For a subset of experiments, T0, T2 and T3 originated from the same biological replicate used in another run, prior to treatment allocation; these time points were therefore not independent.

Table S2. Mortality of *Z. morio* larvae (*Zm*L) during the different experiments and at diverse time points

***Zm*L group**	**Experiment**	**Mortality, No. of *Zm*L (%)**		
		**After giving food contaminated with *Sk*-1 for 3 days (T3) [a]**	**After the first force-feeding (T5) [b]**	**After the second force-feeding (T7) [b]**
**Untreated**	Exp-1	0/64 (0%)	0/20 (0%)	0/16 (0%)
	Exp-2	0/64 (0%)	0/20 (0%)	0/16 (0%)
	Exp-3	0/114 (0%)	0/20 (0%)	0/16 (0%)
	Total	0/242 (0%)	0/60 (0%)	0/48 (0%)
**Phage-treated (*INTESTI*bc)**	Exp-1	0/64 (0%)	0/24 (0%)	2/20 (10%)
	Exp-2	0/64 (0%)	0/23 (0%)	2/15 (13.3%)
	Exp-3	0/114 (0%)	1/19 (5.3%)	2/15 (13.3%)
	Total	0/242 (0%)	1/66 (1.5%)	6/50 (12.0%)
**dPBS-treated**	Exp-1	0/32 (0%)	0/17 (0%)	1/13 (4.5%)
	Exp-2	0/64 (0%)	0/20 (0%)	1/16 (6.3%)
	Exp-3	0/114 (0%)	1/18 (5.6%)	1/14 (7.1%)
	Total	0/210 (0%)	1/55 (1.8%)	3/43 (7.0%)
**OVERALL**		0/694 (0%)	2/181 (1.1%)	9/141 (6.4%)

[a] The numbers indicated refer to the initial number of *Zm*L at T0 before taking at various time points the 4 larvae for analyses. Note also that for a subset of experiments, T0, T2 and T3 originated from the same biological replicate used in another run, prior to treatment allocation; these time points were therefore not independent (see Table S1).

[b] For the untreated group, there was no force-feeding at T3 and T5.

Table S3. Phenotypic characteristics of the 12 representative *Sk*-1 strains recovered during *Zm*L experiments

**Antibiotic**	**MIC, µg/mL (interpretation)** [a]												
	**Original *Sk*-1 (reference)**	***Sk*-1 T7 Exp1 Untreated**	***Sk*-1 T7 Exp2 Untreated**	***Sk*-1 T7+ Exp2 *INTESTI*bc**	***Sk*-1 T7+ Exp3 *INTESTI*bc**	***Sk*-1 T10 Exp1 Untreated**	***Sk*-1 T10 Exp2 Untreated**	***Sk*-1 T10+ Exp2 *INTESTI*bc**	***Sk*-1 T10+ Exp3 *INTESTI*bc**	***Sk*-1 T14 Exp1 Untreated**	***Sk*-1 T14 Exp2 Untreated**	***Sk*-1 T14+ Exp2 *INTESTI*bc**	***Sk*-1 T14+ Exp3 *INTESTI*bc**
Piperacillin-tazobactam	>64 (R)	>64 (R)	>64 (R)	>64 (R)	>64 (R)	>64 (R)	>64 (R)	>64 (R)	>64 (R)	>64 (R)	>64 (R)	>64 (R)	>64 (R)
Ticarcillin-clavulanate	>128 (R)	>128 (R)	>128 (R)	>128 (R)	>128 (R)	>128 (R)	>128 (R)	>128 (R)	>128 (R)	>128 (R)	>128 (R)	>128 (R)	>128 (R)
Cefotaxime	>32 (R)	>32 (R)	>32 (R)	>32 (R)	>32 (R)	>32 (R)	>32 (R)	>32 (R)	>32 (R)	>32 (R)	>32 (R)	>32 (R)	>32 (R)
Ceftazidime	>16 (R)	>16 (R)	>16 (R)	>16 (R)	>16 (R)	>16 (R)	>16 (R)	>16 (R)	>16 (R)	>16 (R)	>16 (R)	>16 (R)	>16 (R)
Cefepime	>16 (R)	>16 (R)	>16 (R)	>16 (R)	>16 (R)	>16 (R)	>16 (R)	>16 (R)	>16 (R)	>16 (R)	>16 (R)	>16 (R)	>16 (R)
Aztreonam	>16 (R)	>16 (R)	>16 (R)	>16 (R)	>16 (R)	>16 (R)	>16 (R)	>16 (R)	>16 (R)	>16 (R)	>16 (R)	>16 (R)	>16 (R)
Imipenem	≤1 (S)	≤1 (S)	≤1 (S)	≤1 (S)	≤1 (S)	≤1 (S)	≤1 (S)	≤1 (S)	≤1 (S)	≤1 (S)	≤1 (S)	≤1 (S)	≤1 (S)
Meropenem	≤1 (S)	≤1 (S)	≤1 (S)	≤1 (S)	≤1 (S)	≤1 (S)	≤1 (S)	≤1 (S)	≤1 (S)	≤1 (S)	≤1 (S)	≤1 (S)	≤1 (S)
Ertapenem	1 (R)	1 (R)	1 (R)	1 (R)	0.5 (S)	1 (R)	1 (R)	1 (R)	1 (R)	1 (R)	0.5 (S)	1 (R)	0.5 (S)
Doripenem	0.25 (S)	0.5 (S)	0.5 (S)	0.5 (S)	0.25 (S)	0.25 (S)	0.25 (S)	0.25 (S)	0.25 (S)	0.25 (S)	0.25 (S)	0.25 (S)	0.25 (S)
Gentamicin	2 (S)	2 (S)	2 (S)	2 (S)	2 (S)	2 (S)	2 (S)	2 (S)	2 (S)	≤1 (S)	2 (S)	2 (S)	2 (S)
Tobramycin	>8 (R)	>8 (R)	>8 (R)	>8 (R)	>8 (R)	>8 (R)	>8 (R)	>8 (R)	>8 (R)	>8 (R)	>8 (R)	>8 (R)	>8 (R)
Amikacin	16 (R)	16 (R)	16 (R)	16 (R)	16 (R)	16 (R)	16 (R)	16 (R)	16 (R)	16 (R)	16 (R)	16 (R)	16 (R)
Levofloxacin	>8 (R)	>8 (R)	>8 (R)	>8 (R)	>8 (R)	>8 (R)	>8 (R)	>8 (R)	>8 (R)	>8 (R)	>8 (R)	>8 (R)	>8 (R)
Ciprofloxacin	>2 (R)	>2 (R)	>2 (R)	>2 (R)	>2 (R)	>2 (R)	>2 (R)	>2 (R)	>2 (R)	>2 (R)	>2 (R)	>2 (R)	>2 (R)
Trimethoprim/sulfamethoxazole	≤0.5 (S)	1 (S)	1 (S)	1 (S)	1 (S)	1 (S)	1 (S)	1 (S)	1 (S)	1 (S)	≤0.5 (S)	≤0.5 (S)	≤0.5 (S)
Colistin	≤0.25 (S)	≤0.25 (S)	≤0.25 (S)	≤0.25 (S)	≤0.25 (S)	≤0.25 (S)	≤0.25 (S)	≤0.25 (S)	≤0.25 (S)	≤0.25 (S)	≤0.25 (S)	≤0.25 (S)	≤0.25 (S)
Polymyxin B	0.5 (NA)	0.5 (NA)	0.5 (NA)	≤0.25 (NA)	≤0.25 (NA)	≤0.25 (NA)	0.5 (NA)	≤0.25 (NA)	≤0.25 (NA)	≤0.25 (NA)	≤0.25 (NA)	≤0.25 (NA)	≤0.25 (NA)
Doxycycline	8 (NA)	8 (NA)	8 (NA)	8 (NA)	8 (NA)	8 (NA)	8 (NA)	8 (NA)	8 (NA)	8 (NA)	8 (NA)	8 (NA)	8 (NA)
Minocycline	8 (NA)	8 (NA)	8 (NA)	8 (NA)	8 (NA)	8 (NA)	8 (NA)	8 (NA)	8 (NA)	8 (NA)	8 (NA)	8 (NA)	8 (NA)
Tigecycline	0.5 (S)	0.5 (S)	0.5 (S)	0.5 (S)	0.5 (S)	0.5 (S)	0.5 (S)	0.5 (S)	0.5 (S)	0.5 (S)	0.5 (S)	0.5 (S)	0.5 (S)
**Susceptibility to *INTESTI*bc** [b]	++++ (S)	++++ (S)	++++ (S)	++++ (S)	++++ (S)	++++ (S)	++++ (S)	++++ (S)	++++ (S)	++++ (S)	++++ (S)	++++ (S)	++++ (S)

[a] According to the EUCAST criteria 2025 (v15): R, resistant; S, susceptible; NA, not applicable or not available. MIC values were obtained implementing only the Sensititre GNX2F plate (Thermo Fisher Scientific).

[b] Susceptibility of *Sk*-1 strains to the *INTESTI*bc as assessed by double-layer agar method (DLA). "++++" denotes complete clearing (confluent lysis).

Table S4. Genome SNVs in recovered *Sk*-1 strains using the original *Sk*-1 as the reference

Genomic location [a]	Position [b]	Original *Sk*-1 (reference)	***Sk*-1 strains isolated during *in vivo* experiments**												Annotations
			T7 Exp1	T7 Exp2	T7+ Exp2	T7+ Exp3	T10 Exp1	T10 Exp2	T10+ Exp2	T10+ Exp3	T14 Exp1	T14 Exp2	T14+ Exp2	T14+ Exp3	
chromosome-*Sk*-1	251256	A	A	G	G	G	A	G	G	G	A	G	G	G	periplasmic copper metallochaperone CueP
	2010865	A	A	A	A	A	A	A	A	A	A	A	T	A	ribosome modulation factor RmF
	2050474	G	G	G	G	G	T	G	G	G	G	G	G	G	4-hydroxyphenylacetate permease HpaX
	2094381	C	C	C	C	C	A	C	C	C	C	C	C	C	glucans biosynthesis glucosyltransferase MdoH
	2329320	C	C	C	C	C	C	C	A	C	C	C	C	C	non-CDS
	2543746	G	G	T	G	T	G	T	G	T	G	T	G	T	virulence-associated effector SrfC
	2707443	G	G	G	G	G	T	G	G	G	G	G	G	G	non-CDS
	3045083	G	G	G	G	G	G	A	G	G	G	G	G	G	putrescine/proton symporter PlaP
Plasmid p1-*Sk*-1 [c]	58689	T	T	T	A	T	T	T	T	T	T	T	T	T	conjugal transfer protein TraW

Note. non-CDS, non-coding sequences.

[a] Genome analysis (alignment = 98.5%)

[b] Position refers to the genome of the original *Sk*-1 strain used as reference (PRJNA1267977)

[c] p1-*Sk*-1 corresponds to the 63,5-Kb IncL plasmid containing the *bla*$_{OXA-48}$.
